# Methylation and hydroxymethylation of CpG display dynamic landscapes in early embryo development and define differentiation into embryonic and placental lineages

**DOI:** 10.1186/1756-8935-6-S1-P60

**Published:** 2013-03-18

**Authors:** Yan Li, Chris O’Neill

**Affiliations:** 1 Centre for Developmental and Regenerative Medicine, Kolling Institute of Medical Research, Sydney Medical School, NSW, 2065, Australia

## 

Covalent modifications to cytosine provide important epigenetic information required for normal embryo development. 5’methylcytosine (5mC) has received most experimental attention while 5’hydroxymethyl cytosine (5hmC) has assumed recent prominence. Extant gold-standard methods of analysis of do not discriminate between 5mC and 5hmC hence most published methylomes have limits to their interpretation. Immunolocalization allows discrimination between the range of modifications, provides a genome-wide level of analysis, allows differential assessment of nuclear localisation, and is compatible with the limited DNA available within the early embryo. Using newly established methodology for full retrieval of these two antigens [1] we have reassessed the patterns of expression of 5mC and 5hmC across preimplantation development. Both the male and the female pronuclei show extensive and relatively stable staining of both 5mC and 5hmC across all stages of zygotic maturation. The analysis does not provide support for the oft claimed active global demethylation of the paternally-derived genome relative to that of the maternal-derived genome, and also provide no support for a role of 5hmC as an intermediate in such an active demethylation step. During the cleavage stage of development (2-cell to 8-cell) mC became progressively more associated with heterochromatic regions of the nucleus while 5hmC staining was the dominant modification in euchromatin. By the morulae stage several cells with inner positions in the embryo showed some reduced euchromatic staining of 5hmC and 5mC. Relative to trophectodermal cells, the pluripotent inner cell mass of blastocysts show a marked overall reduction in euchromatic of both 5hmC and 5mC staining, and a progressive reduction of mC staining from heterochromatic foci. The distinctively different landscapes of 5mC and 5hmC on the embryonic genome indicate that these modifications may provide different epigenetic information to the early embryo, and shows that differential changes in this landscape define the first differentiation events in the early embryo. Current claims of extensive remodelling of CpG in the zygote seem to be largely caused by dynamic changes in the conformation of chromatin in early development leading to extensive antigenic masking of these modifications. When this masking is removed a much greater level of stability of these modifications at a global level becomes evident.
